# High-quality full genome assembly of historic *Xylella fastidiosa* strains from ICMP collection using a hybrid sequencing approach

**DOI:** 10.1128/MRA.00536-23

**Published:** 2023-10-17

**Authors:** Pragya Kant, Natasha Brohier, Rachel Mann, Luciano Rigano, Robert Taylor, Johanna Wong-Bajracharya, Toni A. Chapman, Monica Kehoe, Rebecca Roach, Brendan Rodoni, Fiona Constable

**Affiliations:** 1 Department of Energy, Environment and Climate Action, Agriculture Victoria Research, Bundoora, Victoria, Australia; 2 Plant Health and Environment Laboratory, Ministry for Primary Industries, Auckland, New Zealand; 3 Department of Primary Industries, Plant Biosecurity Research and Diagnostics, New South Wales, Australia; 4 Department of Primary Industries and Regional Development, Biosecurity and Sustainability, Western Australia, Australia; 5 Department of Agriculture and Fisheries, Queensland, Australia; University of Maryland School of Medicine, Baltimore, Maryland, USA

**Keywords:** bacteriology, *Xylella fastidiosa* subspecies *fastidiosa*, *Xylella fastidiosa* subspecies* multiplex*, long and short reads, MinION

## Abstract

High-quality complete genomes of five *Xylella fastidiosa* strains were assembled by combining Nanopore and Illumina sequencing data. Among these, International Collection of Micro-organisms from Plants (ICMP) 8731, ICMP 8742 and ICMP 8745 belong to subspecies *fastidiosa* while ICMP 8739 and ICMP 8740 were determined as subspecies *multiplex*. The strains were further classified into sequence types.

## ANNOUNCEMENT

*Xylella fastidiosa* is a xylem-limited plant pathogenic bacterium that has caused significant epidemics and economic losses in many plant species in the Americas and Europe (1). This bacterium is the number one quarantine pathogen for Australia and New Zealand. The genus *Xylella* has complex taxonomy, having two species: *fastidiosa* and *taiwanensis*. Within the *fastidiosa,* there are six known subspecies, three of them, *fastidiosa*, *multiplex,* and *pauca*, are of significance based on the genomic analysis (2). Furthermore, *X. fastidiosa* is differentiated into sequence types (3).

We obtained DNA from ICMP (International Collection of Micro-organisms from Plants), New Zealand for sequencing. The strain ICMP 8731 was isolated from *Vitis vinifera* and ICMP 8745 from *Ambrosia*, respectively, in Florida. The strains ICMP 8740 and ICMP 8742 were collected from host *Platanus occidentalis* and *Ulmus americana*, respectively, in Washington, DC, and ICMP 8739 was collected from *Prunus dulcis* in California. All bacterial cultures were isolated in the 1980s and had not undergone prior sequencing.

Illumina sequencing library were generated using PerkinElmer NEXTFLEX Rapid XP DNA-Seq Kit at half reaction with PerkinElmer NEXTFLEX UDI Barcodes (PerkinElmer, Massachusetts, USA). Library was paired-end sequenced (2 × 150 bp) using the Illumina NovaSeq 6000. Quality control (QC) of the data were processed using fastp (4). For Nanopore sequencing, Rapid RBK004 library was prepared and sequenced using R9.4 flowcell in a MinION MkIB device (Oxford Nanopore Technologies) following manufacturer’s instructions. The sequencing was controlled with MinKNOW software. Output fast5 files were basecalled and demultiplexed using high-accuracy model of Guppy V6.4.8 to obtain fastq files. NanoPlot (https://github.com/wdecoster/NanoPlot) was used for reads QC.

Hybrid assemblies were performed by Unicycler version 0.4.7 using both Nanopore and Illumina reads.

All *X. fastidiosa* strains were circularized in a chromosome except for ICMP 8739 and ICMP 8745, which were visualized with gaps and nodes using Bandage V0.8.1 (5). Annotations were generated by National Center of Biotechnology Information (NCBI) Prokaryotic Genome Annotation Pipeline (6) (Table 1).

**TABLE 1:** Description and annotation metrics of the five ICMP *Xylella fastidiosa* strains hybrid assemblies

Strain name	ICMP 8731	ICMP 8739	ICMP 8740	ICMP 8742	ICMP 8745
Subspecies	*fastidiosa*	*multiplex*	*multiplex*	*fastidiosa*	*fastidiosa*
BioSample	SAMN33037963	SAMN33037965	SAMN33037964	SAMN33037966	SAMN33037967
GenBank accession	CP117821.1	JAQQTM000000000	CP117820.1	CP117823.1	JAQQTL000000000
Genome size (bp)	2,409,646	2,023,822	2,548,838	2,556,755	2,589,186
Nanopore reads	230,567	281,205	440,928	180,657	70,444
Nanopore N50 (bp)	3,069	2,523	5,225	7,306	6,247
Nanopore coverage	152×	179×	436×	246×	75×
Illumina paired-reads	7,816,992	7,121,802	7,954,171	8,174,227	8,198,756
Total Illumina bases	1,174×	1,283×	1,137×	1,154×	1,147×
GC content	51.53%	51.67%	51.65%	51.49%	51.54%
CDS	2,084	1,711	2,227	2,252	2,308
Genes	2,136	1,765	2,286	2,311	2,367
ncRNA	3	3	3	3	3
Regulatory	1	1	1	1	1
rRNA	3	6	6	6	6
tmRNA	1	1	1	1	1
tRNA	45	44	49	49	49
Plasmid name	pXFAS01			pXF879-41	
Plasmid size (bp)	38,298			41,752	

**Fig 1:**
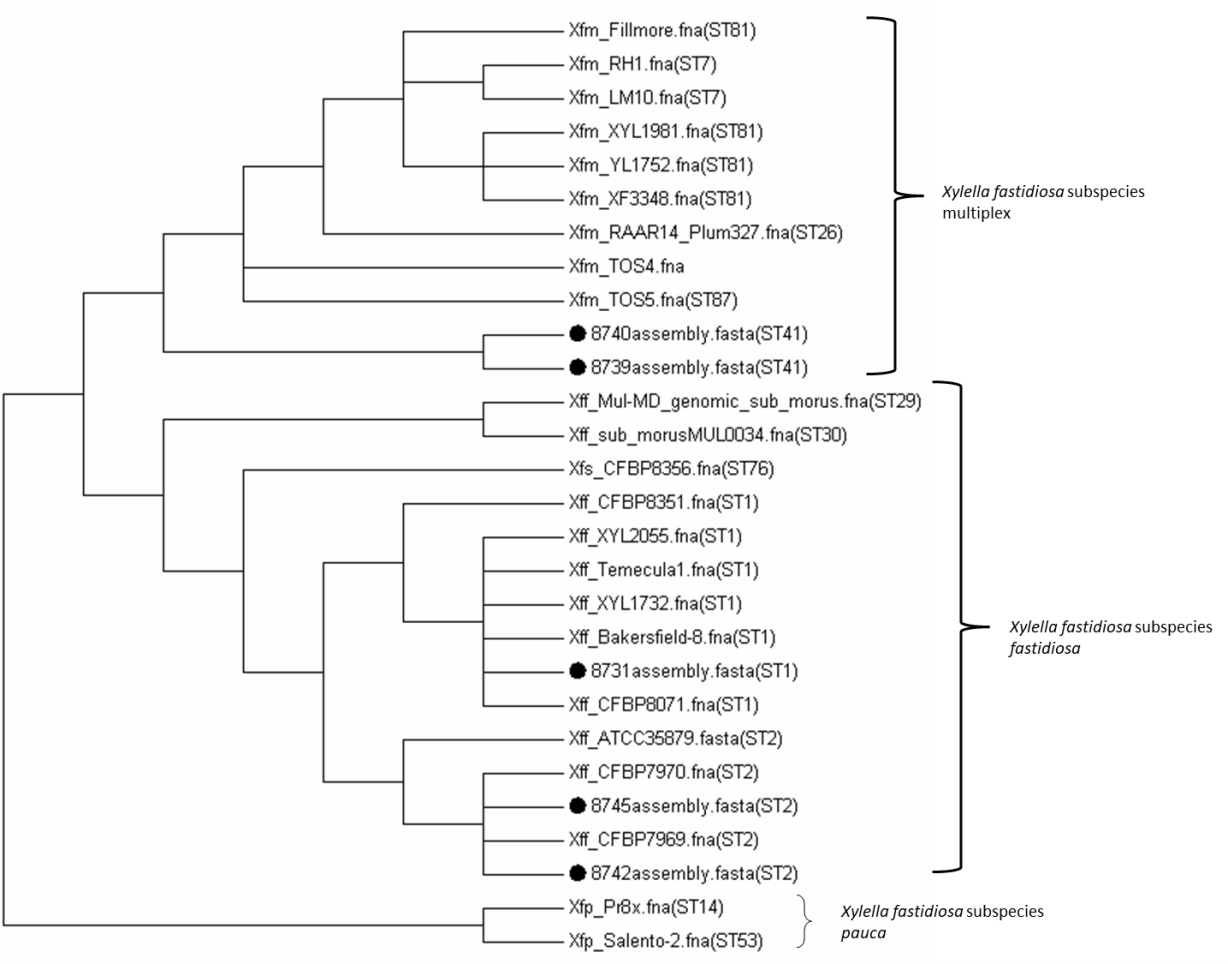
Phylogenetic tree displaying relatedness of the of 29 *Xylella fastidiosa* genomes using UBCG analysis. The tree was visualized in MEGA V7 (https://www.megasoftware.net/). Filled dots represent strains sequenced in this study. Sequence type (ST) is provided in parentheses.

A phylogenetic analysis and a tree were generated using an up-to-date bacterial core gene (UBCG) set, a core genome analysis pipeline that extracts and concatenates 92 core bacterial genes by maximum-likelihood approach (7). In addition, sequence types were determined using Linux-based single script (https://github.com/tseemann/mlst). All five strains were accurately placed to their respective clades in the dendrogram (Fig. 1). For analysis, softwares were used with default parameter, unless specified otherwise.

## Data Availability

Genome assembles are accessible in GenBank as Bioproject- PRJNA930964 consisting five accessions CP117821.1, JAQQTM000000000, CP117820.1, CP117823.1, JAQQTM000000000. The raw reads are deposited in Sequence Read Archive, accession numbers SRR24958443 to SRR24958447 (Illumina) and SRR24955971 to SRR24955975 (Nanopore).
